# Street research market: dealing with scientific misconduct in Iran

**DOI:** 10.1186/s12910-020-00518-x

**Published:** 2020-08-24

**Authors:** Homayoun Sadeghi-Bazargani, Leila Nikniaz, Hamid Reza Yousefi Nodeh

**Affiliations:** 1 Road Traffic Injury Research Center, Department of Statistics and Epidemiology, Tabriz University of Medical Sciences, Tabriz, Iran; 2 Tabriz Health Services Management Research Center, Tabriz University of Medical Sciences, Daneshgah Street, Tabriz, Iran; 3 Student research committee, Research Center for Evidence-Based Medicine, Tabriz University of Medical Sciences, Tabriz, Iran

**Keywords:** Street research, Scientific misconduct, Iran, Legislation

## Abstract

**Background:**

Scientific misconduct is a prevalent phenomenon with many undesirable consequences. In Iran, no original research have been done about scientific fraud. So, this study aimed at describing a challenging research misconduct in Iran, its related causes, and the ways Iranian authorities deal with it.

**Methods:**

In this cross-sectional study, through a two-year period, all the advertisements installed in the study sites were collected and the content analysis was performed. Semi-structured interviews were held with experts for discovering the causes of misconduct. Also, published issues were collected for review of the laws on confronting the fraud in Iran.

**Results:**

The content analysis resulted in identifying four categories of misconduct issues: advertising approach, types of services, outcome guarantee, and justifying the academic credit. Besides, reviewing the related literature indicated that Iranian government and the responsible authorities have recently established serious penalties for dealing with scientific misconduct through legislation.

**Conclusions:**

This study revealed some misconduct in scientific activities which has persuaded the authorities to enforce strict rules to deal with it. The effectiveness of this legislation needs to be investigated in some further studies.

## Background

Misconduct refers to the planned and consciously fabrication, misrepresentation, or plagiarism of scientific research [1]. Scientific misconduct is a prevalent phenomenon that the scholastic organizations have challenged with it over the history of scientific publication and had many undesirable consequences [2]. Several studies in different countries have shown that misconduct is considered as a global issue not restricted to a special country and unfortunately the evidence is growing. Although not all the retractions are due to fraud, a recent review has stated that since 1975, 10-fold increase was shown in retractions of scientific articles due to fraud [2]. Fraud, plagiarism and duplicate publication were attributed to 43.4, 9.8 and 14.2% of cases, respectively. Error was attributable to only 21.3% of cases. Seventy-five percent of fraud related retractions belonged to Japan, China and the United States [3].

Today, with the increasing number of scientists, novel practices of misconduct in the educational systems of the world has existed [4]. Nevertheless, it is less common to recognize the research misconduct practices and the public and governments’ reactions to them in different countries [4]. Recently, a report was published in Science by Stone [5] about specific shops which produced scientific papers particularly for students in Iran which caused different reactions [6]. In this marketing institutions and advertisers offer to write students’ theses and scientific papers for a fee, advertising on the internet or via the placard-carrying touts outside the University. The mentioned report was a visiting case report which showed that a survey is needed to describe the details of this type of scientific fraud in Iran. Thus, with a two-year survey, we described a sample of research misconduct in Iran, its causes and how the Iranian government dealt with it through legislation.

This study described a challenging research misconduct in Iran, its related causes, and the ways Iranian authorities deal with it.

## Methods

### Iranian academic system structure

In Iran, higher education is provided at universities and colleges/institutions. Some of these are private educational institutions (*n* = 37) and the others are State-run institutions offer free education (*n* = 90). University level studies in Iran are divided into three stages, associate’s degree (*Kardani*) or bachelor’s degree (*Karshenasi*), master’s degree (*Karshenasi-arshad*), and doctorate. At the undergraduate level, thesis is not mandatory; however, in some disciplines, it is mandatory to complete a research project. In the postgraduate level, it is necessary to complete the dissertation and defense, except for some fields in Payame Noor University, which does not require a thesis for master’s degree.

In Iran, the National Committee on Ethics in Biomedical Research was established in 1998 in the Research Deputy of the Ministry of Health, and a year later, the Regional Committees on Ethics in Research were established in universities and research centers. All of the universities of medical sciences in Iran have local ethical committees. These committees undertake of the supervision and observation of national and international laws on medical ethics in research. Ethics committees in universities are responsible for investigating research misconduct and providing ethical codes to research projects.

As this study is an exploratory study, we actually used three different sources for collecting the data regarding answering the questions: 1- a field work for demonstrating the status of misconduct 2- Interview with experts for explaining the pattern of this type of misconduct 3- a literature review for finding legislations.

#### Fieldwork

The present study was conducted through a qualitative content analysis from 2015 to 2017. At first, under targeted sampling, top and key universities in Tehran were identified and visited (Amirkabir University of Technology, Iran University of Science and Technology, Islamic Azad University, Islamic Azad University of Medical Sciences, Shahid Beheshti University of Medical Sciences, Sharif University of Technology, Tarbiat Modares University, Tehran University of Medical Sciences, and University of Tehran). Around these universities, number and content of installed advertisements were assessed. Based on the first step and number and content of the installed advertisements, the near locations to Tehran university of medical sciences and university of Tehran were identified as the most suspected area for scientific fraud (also stated in Stone’s report) and was considered for sampling. These universities were located in Enghelab square of Tehran which is a highly frequented area by students because of the presence of some major universities of Tehran, the high number of student dormitories and existence of many well-known bookstores and scientific service delivery institutions. Thus, these sites are usually considered as the best places for installation of advertisements related to scientific activities.

### Sampling

Sampling included eight phases of 45–60 min walks in the predetermined locations in which photographs were taken from the advertisements installed in the study sites. All the advertisements related to translating, data analyzing, article acceptance, writing article, thesis & proposal, book authoring and the other services were enrolled. Some institutions had different forms of advertisements with different contents. All these varying contents were shot and considered.

### Content analysis

As a preliminary analytic step, all ads were examined. Manifest and latent analysis methods were applied to analyse the ads. In manifest analysis, the ads̕ installation location, style, and discipline were analysed and through the latent analysis, the categories of the misconduct problem were identified. In this phase, the duplicate ads were excluded; all the institutions were identified and categorized. For identifying categories, the texts and phrases of posters, banners and advertising cards were thoroughly studied, interpreted and encoded. Codes were developed inductively by reading the advertisements multiple times. The information were categorized (codes with a common concept were categorized under a theme and named). Coding was done by two researchers using the following process: Data immersion (getting familiar with context of data), identifying and extracting primary codes, identifying themes (putting extracted primary codes in related themes), reviewing and completing identified themes, naming and defining themes and assuring the reliability of extracted codes and themes. During the coding process, the two evaluators with previous experience of quality assessment independently assessed the advertisements. In case of disagreement, they worked out a settlement together through discussion. In order to avoid unnecessary repetitions, if an advertisement appeared many times, it was counted as one. Agreement between the coders was verified using The Kappa coefficient (*k*). Values below 0.40 or so represent poor agreement beyond chance, kappa values between 0.40 and 0.75 represent intermediate agreement, and values greater than 0.75 or so represent excellent agreement beyond chance [7].

#### Interview with experts

To address the reasons of scientific fraud in Iran, our study investigated expert views regarding this issue with a questionnaire developed for this study (Additional file 1). The questions were as follows: What is your understanding of scientific fraud? Do you think there are gaps, issues or problems related to scientific integrity in Iran? What do you perceive to be the primary threats to the scientific integrity in Iran? What do you perceive to be the main sources of scientific misconduct in Iran? What do you perceive to be the role of universities in growing of scientific fraud in Iran? What do you perceive to be the role of health ministry in growing of scientific fraud in Iran? What do you perceive to be the role of students in growing of scientific fraud in Iran? What do you perceive to be the solutions to prevent scientific fraud in Iran? What interventions would make the biggest difference to improve scientific integrity in Iran? In conclusion, is there anything else we haven’t discussed that you wish we had?

For recruiting the experts, a purposive sampling method was used. The experts were identified based on work experience, expertise and affiliations, and their publications in different fields of science. Fifteen experts were contacted by telephone. Also, an additional group of experts (5 experts) was contacted through snowball sampling. Data collection continued until no new themes arose from the data. The interviews were audio-recorded and transcribed verbatim by two researchers. For further strengthening the methodology, multiple coding and validity checks were performed. The transcribed interviews were verified for validity against the recording by HYN, HSB and experts. Disagreements in coding between the researcher and experts were discussed between the two authors (LN and HSB) and experts resulting in final coding of themes. The inter-rater reliability among the researchers who performed the coding was performed and the percentage of disagreement was calculated for each of the interviews.

#### Legislation literature review

The published studies until March 2018 were collected for literature review of the laws on preventing and confronting the fraud in preparation of scientific works in Iran by searching PubMed/MEDLINE, Scopus, EMBASE, Cochrane library, ISI Web of science, Proquest and Google scholar. In addition, Iranian databases including Scientific Information Database, Iranian Research Institute for Information Science and Technology, Magiran, IranMedex, and Barakat knowledge network system, and National System of Laws and Regulations of the Islamic Republic of Iran were searched. The search keywords included (but not limited to): “(law OR legislation OR penalty) AND (minister of science OR president OR speaker of parliament OR government) AND (scientific misconduct OR fraud OR fabrication OR misrepresentation OR plagiarism) AND (Iran)”. The following information was subtracted from the studies: name of the law, approval date, the rules, and the consequences of non-compliance with the laws.

### Ethical issue

Off the different sources for collecting the data, interview with experts was the only part involving human participants. This part of the study was in accordance with the ethical standards of the Ethics Committee of Tabriz University of Medical Science (R.TBZMED.VCR.REC.1398.271) and with the 1964 Helsinki declaration and its later amendments or comparable ethical standards. Also, a written informed consent was obtained from all individual participants included in the study.

## Results

### Fieldwork

In this study, 124 advertisements belonging to 40 advertisers (so called institutions) were identified over eight quarterly sampling runs in a two-year period. Inter-rater reliability was assessed by Pearson’s correlation coefficient, reaching an *r* value of 0.8. The agreement between both observers was also good (Kappa = 0.76). After content analysis of the data, the three categories of misconduct including types of services, outcome guarantee, and justifying the academic credit were defined. In addition, one other category named as “unprofessional advertising approach” is explored that provided beneficial information but not classifiable as misconduct. Table 1 describes the identified categories including the text and phrases applied in the posters, banners and advertising cards. .

**Table 1:** The identified categories (the text and phrases of posters, banners and advertising cards)

Categories	N (%)	Subcategories	Explanations and Quotations	N (%)
**Unprofessional advertising approach (**not classifiable as misconduct**)**	50 (40.3)	Using ambiguous terms	“ISI paper” (refer to Fig. 1.a): what does it mean? Finding ISI papers for literature review of thesis OR providing ISI articles	22 (16.9)
		Improper location for installing ads	Installation of ads on the Relief Committee Fund or trash bins. Refer to Fig. 1.b	21 (16.1)
		Improper installation	Improper installation of ads with sticks. Refer to Fig. 1.a	19 (15.3)
		Unreasonable acceptance time	“Acceptance in 2 days” (Fig. 1.d)	18 (10.8)
		Multiple advertising forms	Darolfonoon Institute with different advertising forms, each for special purposes. Refer to Fig. 1. a & d	14 (11.3)
		Destroying scientific identity	Refer to Fig. 1. a, b	10 (8.0)
**Services**	118 (95.1)	Performing students’ projects and research	Refer to Fig. 1. f	100 (80.6)
		consulting	Refer to Fig. 1. e	89 (71.8)
		Translating	Refer to Fig. 1. e	55 (44.3)
		Printing and typing	Refer to Fig. 1. e	36 (29.0)
		Data analyzing	Refer to Fig. 1. g	42 (33.9)
		Getting an acceptance	Refer to Fig. 1. g	42 (33.9)
		Editing	Refer to Fig. 1. f	31 (25.0)
		Holding educational courses	Refer to Fig. 1. c	25 (20.1)
		Writing articles, theses and proposals	Refer to Fig. 1. f	19 (15.3)
		Book authoring	Refer to Fig. 1. g	11 (8.8)
		Consulting with professors	Refer to Fig. 1. e	10 (8.0)
		Submitting	Refer to Fig. 1. g	8 (6.4)
		Debugging of thesis	Refer to Fig. 1.h	2 (1.6)
		Rewriting	Refer to Fig. 1. g	1 (0.8)
		Thesis related services	Refer to Fig. 1. d	100 (80.6)
**Guarantee**	95 (76.6)	ISI article	Refer to Fig. 1.a	87 (70.2)
		Guarantee	“100% guarantee” refer to Fig. 1.a	23 (18.5)
		Best quality guarantee	“Performing project with best quality and 100% guarantee”. Refer to Fig. 1.i	14 (11.3)
		Free of charge article guarantee bonus	Refer to Fig. 1.j	8 (6.4)
		Services without dealer	Refer to Fig. 1.e	8 (6.4)
		Impact factor guarantee	Refer to Fig. 1.k	3 (2.4)
		Acceptance guarantee	“Get paid after acceptance”. Refer to Fig. 1.a	2 (1.6)
		Time guarantee	“Acceptance or writing article in 5 days”. Refer to Fig. 1.a	1 (0.8)
**Justification**	28 (22.5)	Approved by the Ministry of Science	Refer to Fig. 1.g	3 (2.4)
		Approved by professors	Refer to Fig. 1.e	14 (11.3)
		Approved by PhD students	Refer to Fig. 1.e	8 (6.4)
		Applying well known publisher logos	Refer to Fig. 1.h	5 (4.0)
		Name of the institute	“Amiran Scientific, Educational and Research Institute”. Refer to Fig. 1. c	2 (1.6)

#### Unprofessional advertising approach explore

In this study, 40.3% of advertisements suffered from unprofessional advertising approach which comprises six subcategories as follows: using ambiguous terms, improper location for installing ads, improper installation, unreasonable acceptance time, multiple advertising forms, and destroying scientific identity (Table 1).

**Fig. 1:**
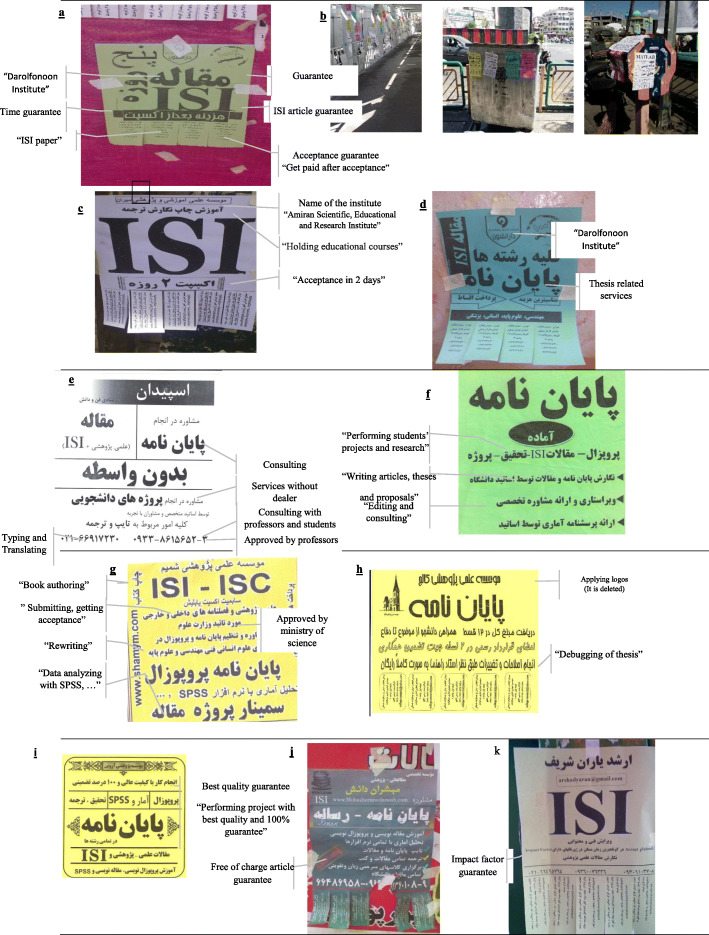
The advertising approach and content of advertisements

Figure 1 illustrates the advertising approach and content of advertisements. As clarified in Fig. 1, ambiguous terms were used in the ads such as “ISI paper” especially for bypassing the potential problems (Fig. 1a). Installation of ads outside the academic environments such as on the trash bins, pillars of the footbridges or relief committee fund boxes (Fig. 1b), and abusing of words such as “Acceptance from ISI journal in 2 days” (Fig. 1 c) were different features of destroying scientific identity. In addition, these ads were improperly and irregularly designed and installed (Fig. 1.a) and 11.3% of them had multiple advertising forms (fig. a & d).

#### Types of services

As indicated in Table 1, 95.1% of advertisements covered different types of services related to misconduct including performing students’ projects and research (Fig. 1.f), consulting (Fig. 1.e), translating (Fig. 1.e), printing & typing (Fig. 1.e), data analyzing (Fig. 1.g), article acceptance (Fig. 1.g), editing (Fig. 1.f), holding research-related courses (Fig. 1.c), writing article, thesis & proposal (Fig. 1.f), book authoring (Fig. 1.g), consulting with professors (Fig. 1.e), submitting (Fig. 1.g), debugging of thesis (Fig. 1.h), rewriting (Fig. 1.g), and thesis related services (Fig. 1.d).

The majority of ads (90.6%) featured two or more services and only 9.3% of them offered a single service. The subcategory entitled ‘performing students’ projects & research’ was the most commonly advertised service (80.6%), followed by ‘consulting’ (71.8%), ‘translating’ (45.2%), and ‘printing & typing’ (44.3%).

#### Outcome guarantee

In this study, 76.6% of the ads had the statement which guaranteed some services such as guarantee of ISI acceptance of the articles (Fig. 1.a), guarantee, used as a single word (Fig. 1.a), best quality guarantee (Fig. 1.i), free of charge article guarantee bonus (Fig. 1.j), services without dealer (Fig. 1.e), guarantee of accepting in high impact factor journal (Fig. 1.k), acceptance guarantee and improvement guarantee (Fig. 1.a). Acceptance in the ISI journals was the most commonly advertised guarantee (70.2%).

#### Justifying the academic credit

In 22.5% of these ads, titles such as “Approved by the Ministry of Science” (Fig. 1.g) or “Approved by professors” (Fig. 1.e), logos such as “Elsevier logo” (Fig. 1.h) and an academic name such as “[name of the institutions] Scientific, Educational and Research Institute” (Fig. 1.c) were applied for justifying the company’s scientific credibility.

### Interview with experts

We selected 20 experts fifteen of whom responded to our original invitation for an interview and the other five were identified by snowball sampling. The response rate was very high (100%). All of the experts were interviewed through 1-h face to face meetings. All transcripts were checked for validity by HYN, HSB and experts. These checks did not result in any significant content changes. The inter-rater reliability was high; on average per interview 78% of the coded items were comparable between the two researchers and experts.

**Fig. 2:**
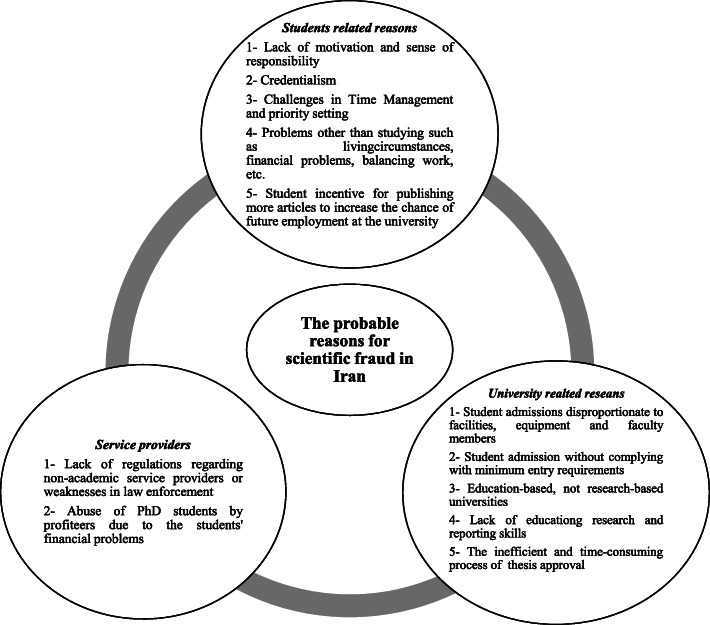
Hypotheses proposed for explaining the reasons for scientific fraud in Iran

The hypotheses proposed for explaining the potential reasons for scientific fraud in Iran is depicted in Fig. 2. Three hypotheses are proposed for this issue including university related reasons, students related reasons and service providers related causes. For each hypothesis, the different subthemes were identified which have been mentioned in Fig. 2.

### Legislation literature review

The search strategy resulted in one criminal law including the single article and ten notes enacted at the plenary session of Islamic Parliament on Tuesday, September 22, 2017, and approved by the Guardian Council on August 30, 2017. The Islamic Parliament enacted “The law on preventing and confronting the fraud in preparation of scientific works”. For summarizing the law, the notes were presented in the supplemental document; however, the content of the Law of Preventing and Confronting the Fraud in Preparation of Scientific Works is summarized as follows:

It is a criminal action to prepare, present, transform works including dissertations, theses, papers, research projects, books, reports or other written or recorded research-scientific works or artistic pieces, whether in electronic or non-electronic format, by any real person or legal entity in order to make a profit and as an occupation -with the aim of presenting the whole work or part of it by someone else as their own works. The perpetrator(s) have to return the earned money to the state treasury and will additionally be penalized as follows:

1. Committing a crime by a real person is subject to a third-grade cash penalty and a sixth-grade deprivation from social rights.
2. In the case of committing a crime by a legal entity, in addition to the punishment of perpetrator(s), managers and the involved coordinators -the penalty of the legal entity is determined in the Islamic Penal Code approved on (April 21, 2013).

## Discussion

Scientific fraud is a universal and multifaceted problem and identification of its patterns, risks and potential solutions are still in its initial stages [8]. Similar to many countries in the world, Iran also suffers from scientific fraud. The authors prefer to use the term “street advertising for research” for this type of fraud. In the current study, we described the details of this type of scientific fraud and how Iranian authorities deal with it.

Based on the results, the approach of street advertisements was inappropriate. Improper use of words, phrases, unsuitable location for installing ads, improper installation and exaggerated claims make these ads far away from principles of advertising [9]. that can result in destroying scientific identity. High demand for scientific fraud is one of the reasons for improper advertising. As the supply is tailored to the demand, the cause of the demand should be critically analyzed. Based on the results of this study, performing students’ projects & research (80.6%) was the most commonly advertised service. Other highly advertised services such as consulting’, ‘translating’, and ‘printing & typing’ were not or less likely for misconduct and increasing the delivery of these services in universities can prevent college students from visiting non-academic institutions. So, the boundary between legitimate publishing support and illegitimate practices should be clarified for the students (researchers) and advertisers to prevent the misconduct.

Based on the expert panel review, three hypotheses are proposed for explaining the potential reasons for scientific fraud in Iran including university related reasons, students related reasons and service providers related causes. So, in providing solutions to prevent scientific fraud, all these aspects must be taken into account.

Legislation is one of the main solutions to prevention and suppression of scientific fraud [10].

However, it seems that the existing legislations are disproportional to the harm caused by misconduct. During the past decade, for prevention of misconduct, only two criminal legal rules were identified to be used. The False Statements Statute, 18. U.S.C. 1001 and The Mail Fraud Statute, 18. U.S.C. 1341 are the examples of the rules [11].

Hopefully, Iran has established ethical committees countrywide to deal exactly with the concerns mentioned in this survey. The new specific law against academic misconduct in Iran is a significant and helpful step toward an improved academic atmosphere [12]. Providing criminal sanctions for scientific misconduct have direct and indirect scientific and public aids. Stimulating researchers to be more cautious in their research is one of the important benefits of criminalizing misconduct [10].

This new law in Iran has punitive point of view to the scientific fraud. Having a visible structure of law implementation and punishment is more effective than having no system [13]. However, it seems that in order to have an effective legislation, punishment should not be the sole legal intervention to address the problem and promoting standards and supportive vision should be considered. In addition, the ways beyond approved legislation such as providing oversight standards for educational and research organizations aimed at controlling the process of performance of the academic practices is suggested. Also, various educational courses such as holding conferences and seminars are needed in order to empowering the technological aspects of the observers and students for prevention of academic misconducts. Moreover, promoting public attitude and knowledge about the development of knowledge is essential and the media has an important role in this regard. Creating the infrastructure needed to train the scientists is the other solution for preventing misconduct and fraud.

This study had some limitations. In this study, all regions of the country were not surveyed and only the areas surrounding the major universities of Tehran were investigated. In addition, it should be noted that internet advertising was not included in this study; however, at the time of performing this research and before establishment of the new rules, there was a few internet advertisements. Although the comprehensive pattern of the problem may vary to some extent taking into account the generalizability of the issue, the explored situation is informative enough for managing the problem. By adopting new laws in Iran and prohibiting street advertising, online advertising may be considered by defrauders. So, constant monitoring and controlling is desired. As expert interview is not an adequate method to identify causality, more precise studies are desired to find the exact causes of misconduct.

## Conclusion

Iran encountered a type of scientific misconduct which might lead to quality loss of scientific productions, competence inequality, and destruction of the scientific identity. The etiologic depiction of the issue can be defined as 12 items in three domains of university related reasons, students related reasons and service providers related causes. For preventing and confronting the fraud, severe impose policies were established by Iranian government. As the survey in this study was conducted before the legislation, future studies should focus on the effectiveness of these rules in preventing scientific misconduct.

## Supplementary information

**Additional file 1.** The interview guide. Supplementary material, doc.

## Data Availability

The datasets used and/or analyzed during the current study are available from the corresponding author on reasonable request.

## Abbreviations

LMIC: Low and Middle Income Countries
Fig: Figure

## References

[1] Buzzelli DE (1993). The definition of misconduct in science: a view from NSF. Science..

[2] Gross C (2016). Scientific misconduct. Annu Rev Psychol.

[3] Fang FC, Steen RG, Casadevall A (2012). Misconduct accounts for the majority of retracted scientific publications. Proc Natl Acad Sci.

[4] Pellegrini PA (2018). Science as a matter of honour: how accused scientists deal with scientific fraud in Japan. Sci Eng Ethics.

[5] Stone R (2016). In Iran, a shady market for papers flourishes. American Association for the Advancement of Science.

[6] Larijani B, Niaz K, Pourabbasi A, Khan F, Spoor J, Abdollahi M (2017). Not only Iranian rise in science marred by fraud: misconduct is a global problem. EXCLI J.

[7] McHugh ML (2012). Interrater reliability: the kappa statistic. Biochemia medica: Biochemia medica.

[8] Pepitone K, Kamat S (2015). A potential solution for research misconduct. JAMA internal medicine.

[9] Frolova S (2014). The role of advertising in promoting a product.

[10] Sovacool BK (2005). Using criminalization and due process to reduce scientific misconduct. Am J Bioeth.

[11] Kline S (1993). Scientific misconduct: a form of white coat crime. J Pharmacy & L.

[12] Ataie-Ashtiani B (2016). Curbing Iran's academic misconduct. Science..

[13] Hesselmann F, Graf V, Schmidt M, Reinhart M (2017). The visibility of scientific misconduct: a review of the literature on retracted journal articles. Curr Sociol.

