# Supplementary material for: Street research market: dealing with scientific misconduct in Iran
Source: BMC Med Ethics. 2020 Aug 24;21:78. doi: 10.1186/s12910-020-00518-x (PMC7446204; doi:10.1186/s12910-020-00518-x)
Supplement: Supplementary file 1 — Additional file 1. The interview guide. Supplementary material, doc. [file 12910_2020_518_MOESM1_ESM.docx]

**
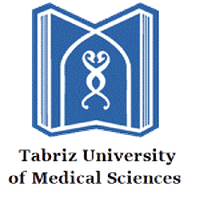
**

**Interviewer:**

**Candidate:**

**Position Title:**

**Date:**

**A PARTICIPATORY QUALITATIVE STUDY OF**

**The reasons and solutions of scientific fraud in Iran**

INTERVIEW GUIDE:

Introduction: As you've heard recently, illegal sales of research, thesis and assignments have been reported in our country. Findings of the first phase of our study showed that there are illegal centers that do all or part of student assignments, projects, or thesis in exchange of money, that is to say selling the research. In the second phase we want to do an online survey in this area to further investigate the causes of the problem and appropriate measures to prevent and diminish the extent of issue.

Questions:

I am going to ask you questions about the reasons of scientific fraud in Iran. There is no right or wrong answer. Please answer each question in detail. Your truthful answer will help the ministry of health to understand the reasons and find the solutions in the country.

1. What is your understanding of scientific fraud?
2. Do you think there are gaps, issues or problems related to scientific integrity in Iran?
3. What do you perceive to be the primary threats to the scientific integrity in Iran?
4. What do you perceive to be the main sources of scientific misconduct in Iran?
5. What do you perceive to be the role of universities in growing of scientific fraud in Iran?
6. What do you perceive to be the role of health ministry in growing of scientific fraud in Iran?
7. What do you perceive to be the role of students in growing of scientific fraud in Iran?
8. What do you perceive to be the solutions to prevent scientific fraud in Iran?
9. What interventions would make the biggest difference to improve scientific integrity in Iran?
10. In conclusion, is there anything else we haven’t discussed that you wish we had?
